# 

**Published:** 1910-12

**Authors:** 


### *The Red Back's Christmas Greeting to its readers*

*"IF in the past, the years have brought*
*Not all the peace and joy you sought,*
*May every one that comes from hence,*
*Bring you some ample recompense.*

*For you deserve so much return,*
*For your heart's treasure nobly spent,*
*The debt should back to you be paid*
*By Love, for what you gladly lent."*

